# Advancements in tissue engineering for cardiovascular health: a biomedical engineering perspective

**DOI:** 10.3389/fbioe.2024.1385124

**Published:** 2024-05-31

**Authors:** Zahra-Sadat Razavi, Madjid Soltani, Golnaz Mahmoudvand, Simin Farokhi, Arian Karimi-Rouzbahani, Bahareh Farasati-Far, Samaneh Tahmasebi-Ghorabi, Hamidreza Pazoki-Toroudi, Hamed Afkhami

**Affiliations:** ^1^ Physiology Research Center, Iran University of Medical Sciences, Tehran, Iran; ^2^ Department of Mechanical Engineering, K. N. Toosi University of Technology, Tehran, Iran; ^3^ Department of Electrical and Computer Engineering, University of Waterloo, Waterloo, ON, Canada; ^4^ Centre for Sustainable Business, International Business University, Toronto, ON, Canada; ^5^ Student Research Committee, USERN Office, Lorestan University of Medical Sciences, Khorramabad, Iran; ^6^ Department of Chemistry, Iran University of Science and Technology, Tehran, Iran; ^7^ Master of Health Education, Research Expert, Clinical Research Development Unit, Emam Khomeini Hospital, Ilam University of Medical Sciences, Ilam, Iran; ^8^ Nervous System Stem Cells Research Center, Semnan University of Medical Sciences, Semnan, Iran; ^9^ Cellular and Molecular Research Center, Qom University of Medical Sciences, Qom, Iran; ^10^ Department of Medical Microbiology, Faculty of Medicine, Shahed University, Tehran, Iran

**Keywords:** bio scaffold, cardiovascular disease (CVD), myocardial infarction (MI), cardiac tissue, tissue engineering

## Abstract

Myocardial infarction (MI) stands as a prominent contributor to global cardiovascular disease (CVD) mortality rates. Acute MI (AMI) can result in the loss of a large number of cardiomyocytes (CMs), which the adult heart struggles to replenish due to its limited regenerative capacity. Consequently, this deficit in CMs often precipitates severe complications such as heart failure (HF), with whole heart transplantation remaining the sole definitive treatment option, albeit constrained by inherent limitations. In response to these challenges, the integration of bio-functional materials within cardiac tissue engineering has emerged as a groundbreaking approach with significant potential for cardiac tissue replacement. Bioengineering strategies entail fortifying or substituting biological tissues through the orchestrated interplay of cells, engineering methodologies, and innovative materials. Biomaterial scaffolds, crucial in this paradigm, provide the essential microenvironment conducive to the assembly of functional cardiac tissue by encapsulating contracting cells. Indeed, the field of cardiac tissue engineering has witnessed remarkable strides, largely owing to the application of biomaterial scaffolds. However, inherent complexities persist, necessitating further exploration and innovation. This review delves into the pivotal role of biomaterial scaffolds in cardiac tissue engineering, shedding light on their utilization, challenges encountered, and promising avenues for future advancement. By critically examining the current landscape, we aim to catalyze progress toward more effective solutions for cardiac tissue regeneration and ultimately, improved outcomes for patients grappling with cardiovascular ailments.

## 1 Introduction

Nearly half of all non-communicable disease-related deaths occur as a result of cardiovascular disease (CVD) (Gao et al., 2021; Gao X. et al., 2022; Bing et al., 2022; Yang et al., 2023). It is essentially caused by a diminished or blocked blood supply to a portion of the heart, resulting in the putrefaction of the cardiovascular muscles. Acute myocardial infarction is one of the most common cardiac emergencies and carries significant morbidity and mortality potential (Dai et al., 2019; Zhao et al., 2020; Gomes et al., 2022; Zhou Y. et al., 2023). It is estimated that AMI can destroy up to 25 percent of the cardiomyocytes (CMs) in the left ventricle, or one billion cells (Salem et al., 2022). There are multiple studies showing that adults have a limited and clinically negligible capacity to renew CMs; as a result, there is not enough replacement tissue formation to compensate for CM loss (Kikuchi et al., 2022). The complication of MI is heart failure (HF), which can be present at the time of admission or during hospitalization. A number of factors contribute to the prevalence of HF, including the aging population and the inability of the heart to regenerate (Williams et al., 2022). The only effective treatment for end-stage heart failure is whole heart transplantation, despite its numerous complications. Due to the limited number of cardiac donors, alternative methods of heart regeneration are urgently needed (Fang et al., 2022). Technology has advanced in recent years, enabling new therapeutic options to emerge. There are now effective regenerative treatments available in preclinical models and clinical trials that offer promise for replacing damaged myocardium (Madonna et al., 2016; Hashimoto et al., 2018; Zhu and Cheng, 2021; Sun B. et al., 2023). Three-dimensional tissue printing, cardiac tissue engineering, and stem cell patches are among these novel options (Cox-Pridmore et al., 2022; Kim et al., 2023). Bifunctional materials and biomaterial matrices have shown promise as replacement materials and reinforcement materials for cardiovascular tissue. Also, stem cells and biomaterial scaffolds can be used in cardiac tissue engineering, which builds tissue constructs *in vitro* for drug screening or implantation (Schmitt et al., 2022; Fu et al., 2023).

**FIGURE 1:**
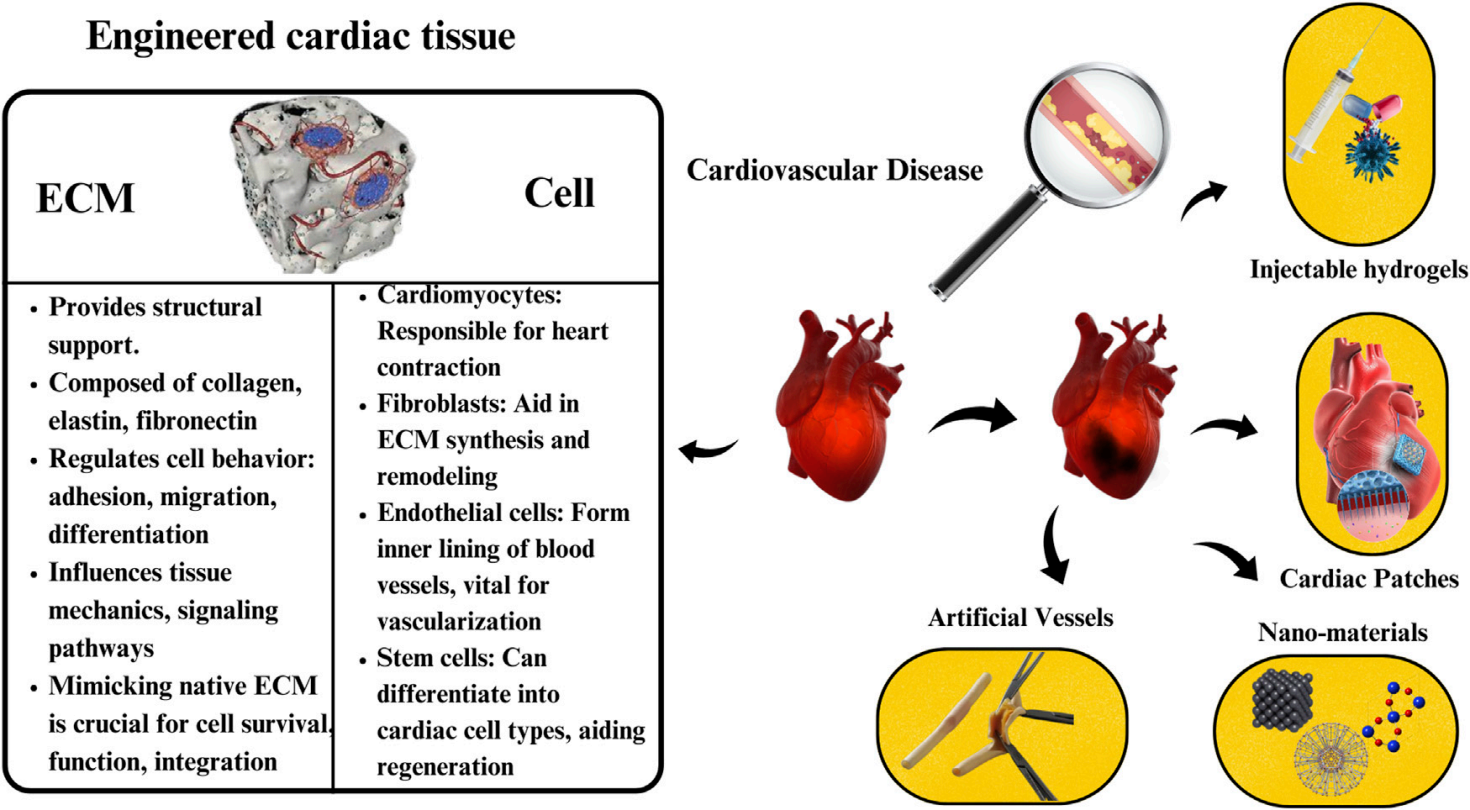
Diverse biomaterials driving innovation in cardiac tissue engineering: from engineered cardiac tissue to injectable hydrogels, patches, nanomaterials, and artificial vessels. ECM: Extracellular Matrix.

By delving into the utilization, challenges, and promising avenues for future advancement of these scaffolds, we provide a thorough analysis of the current landscape in this field. Our focus on how biomaterial scaffolds can address the deficit in cardiomyocytes post-myocardial infarction, and ultimately contribute to improved outcomes for patients with cardiovascular ailments, highlights the novel approach we bring to the discussion. Through this review, we aim to catalyze progress towards more effective solutions for cardiac tissue regeneration, emphasizing the importance of innovative bio-functional materials in advancing the field of cardiac tissue engineering. Figure 1 illustrates the potential impact of biomaterials on cardiovascular health.

## 2 Introduction to tissue engineering in cardiac applications

### 2.1 Understanding acute coronary syndrome

**FIGURE 2:**
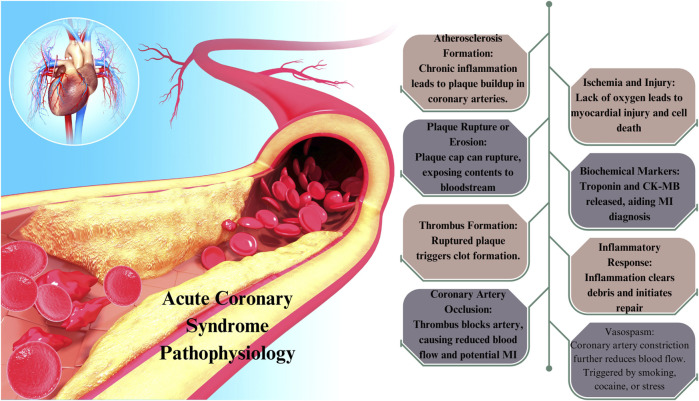
Pathophysiology of acute coronary syndrome (ACS).

Cardiac biomarkers are released into the circulation in response to cardiac or stress-induced harm to the body. The diagnosis of ACS and cardiac ischemia can be facilitated by the utilization of biomarkers, which are indicative of insufficient blood flow in the coronary arteries. Furthermore, cardiac biomarker tests have the potential to not only assess an individual’s susceptibility to ACS and cardiac ischemia, but also to effectively oversee and regulate the care of patients who exhibit indications of ACS or cardiac ischemia. Atherosclerosis, characterized by arterial stiffening and the accumulation of plaque in the artery walls, is well recognized as the primary etiology of Acute Coronary Syndrome (Fazal et al., 2021) and cardiac ischemia (Aksu et al., 2023). As a result, the coronary arteries may undergo stenosis or abrupt occlusion, leading to the constriction of the arteries that provide blood to the heart. Cardiac ischemia occurs as a consequence of insufficient blood flow to the heart. The manifestation of dyspnea, diaphoresis, and angina might ensue as a consequence of inadequate myocardial perfusion. The progressive narrowing of the coronary arteries is a common occurrence in individuals experiencing angina. In the present state, the sensation of pain begins during periods of physical activity and intensifies in tandem with an elevated heart rate. Rapid relief can be achieved by rest or the use of drugs that enhance cardiac blood flow, such as nitroglycerin (Cheema et al., 2012). Atherosclerosis is mostly attributed to the rupture of plaque in acute coronary syndrome (Fazal et al., 2021). When a plaque ruptures, it leads to the formation of a blood clot, also known as a thrombus, within the coronary artery. This occurrence results in an abrupt reduction in both blood flow and oxygen supply to the heart. Episodes of unstable angina may manifest either during periods of rest or persist despite attempts to alleviate symptoms through rest or administration of nitroglycerin, indicating a rapid reduction in blood supply to the heart (Figure 2) (Hatakeyama, 2019).

### 2.2 Utilizing scaffold in cardiovascular disease (CVD) treatment

**FIGURE 3:**
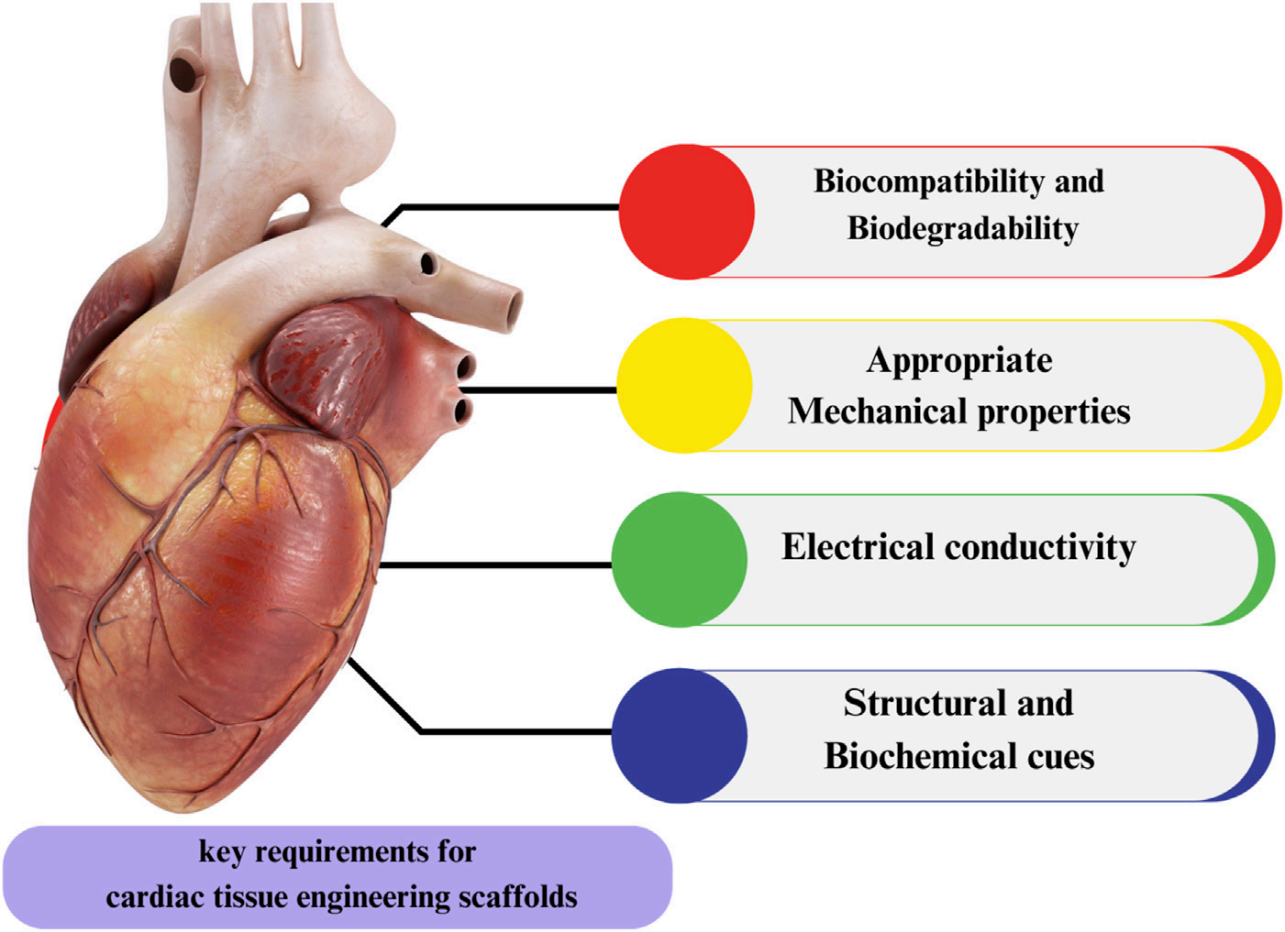
Aspects of scaffold which consider in tissue engineering.

Within the realm of cardiovascular disease, scaffolds play a crucial role in the field of cardiac tissue engineering by serving as a foundational structure that facilitates the seeding of cells and fosters the process of tissue regeneration (Roacho-Perez et al., 2022). The purpose of scaffolds is to facilitate the development of a biomimetic setting that closely replicates the physiological conditions of the heart. This environment is designed to enhance the adhesion of cells and promote the formation of tissue. Scaffolds can be categorized into two distinct classes according to their source: natural and synthetic. Natural materials have a higher degree of biodegradability and biocompatibility, but synthetic materials provide enhanced manipulation of scaffold characteristics (Nikolova and Chavali, 2019). The material properties of scaffolds utilized in cardiovascular tissue engineering must exhibit robust characteristics, given the strength, flexibility, and durability demanded by the circulatory system. Scaffolds serve a vital function in facilitating the delivery of oxygen and nutrients to developing tissue, as well as the removal of metabolic wastes (Dixon and Gomillion, 2022). Additionally, they aid in the assimilation of the designed tissue into the vascular system of the host. Bioresorbable scaffolds, specifically bioresorbable vascular scaffolds (BVS), present a promising therapeutic strategy in the management of coronary artery disease. The scaffolds undergo a slow dissolution process, facilitating the regeneration of native tissue and obviating the necessity for permanent implants. Biomaterials, including bioresorbable polymers, are utilized in the fabrication of scaffolds for cardiovascular applications (Peng et al., 2020). These biomaterials possess the potential to address the constraints associated with existing therapies for coronary heart disease. The optimal characteristics of a scaffold for cardiac tissue engineering involve enabling the development of mature contractile phenotypes in cardiomyocytes and promoting effective intercellular communication with neighboring cells. The longevity of injectable cells is limited by the absence of adaptable three-dimensional features and insufficient nutrition input from the extracellular matrix (ECM) (Majid et al., 2020a). As a consequence, it has been shown that a significant proportion of cells in the human body, approximately 90%, undergo cell death due to inadequate food supply, as well as exposure to free radicals and inflammatory cytokines (Ciolacu et al., 2022). The utilization of a density gradient centrifuge is employed for the purpose of segregating stem cells (SCs) from bone marrow in the context of cell therapy. The outcome of this process yields bone marrow mononuclear cells (BMMNCs), which encompass hematopoietic stem cells (HSCs), bone marrow mesenchymal stem cells (MSCs), and cells that have committed to certain phases of development. Most clinical trials that have used bone marrow-derived mononuclear cells (BMMNCs) have demonstrated moderate enhancements in left ventricular function and cardiac perfusion (Cox-Pridmore et al., 2022; Yu et al., 2022). In addition to adipose tissue and umbilical cord blood, alternative sources of stem cells can also be utilized for extraction. The intricate nature of the anatomical composition of enlarged cardiac organs presents significant difficulties in the process of their accurate evaluation. Dynamic cardiac tissue is formed by the repetitive contraction and relaxation of heart tissue (Amin et al., 2020). The myocardium of native origin has a hierarchical structure, which poses challenges in terms of manipulation. The autonomous contraction of a blood pump is sustained by the orchestrated arrangement of certain extracellular matrix (ECM) proteins and cells. Consequently, it is imperative to employ specialized tissue engineering techniques that can offer scaffolds integrated with a biologically derived extracellular matrix (ECM) to effectively augment and sustain the contractile mechanisms (Figure 3) (Da Silva et al., 2020).

## 3 Types of scaffolding in cardiac tissue engineering

Scaffolding of various forms is essential in cardiac tissue engineering to accurately replicate the natural myocardium for therapeutic purposes. These scaffolds are specifically engineered to mimic the structural, biochemical, mechanical, and electrical characteristics of cardiac tissues. Various categories of scaffolds encompass synthetic scaffolds, such as hydrogels, which possess attributes like non-linear elasticity, anisotropy, and viscoelasticity, closely approaching the features of the myocardium and cardiac valves. Cellulosic scaffolds with 3D origami crease patterns have been created to enhance cell alignment and facilitate the directed contraction of heart tissue (Sun L. et al., 2023; Huang et al., 2023). Additionally, researchers are investigating the impact of micro scaffold patterns such as micropillar, microchannel, cross-linked grid, interwoven, honeycomb, elliptical, and sponge-like structures on the mechanical, porosity, and elasticity properties of cardiac scaffolds. The presence of various scaffold types is crucial for facilitating cell adhesion, migration, differentiation, and proliferation in the field of cardiac tissue engineering (Table 1) (Zhou J. et al., 2023).

**TABLE 1:** Application of most common scaffold and characteristics.

Scaffolding type	Description	Characteristics	Examples and applications
Natural Polymers	Derived from natural sources (e.g., collagen, fibrin, gelatin)	Biocompatible, bioactive, mimic native ECM, provide biochemical cues for cell adhesion and growth	Collagen: Supports cardiomyocyte alignment and function. Fibrin: Promotes angiogenesis. Gelatin: Enhances cell adhesion and proliferation
Synthetic Polymers	Man-made polymers (e.g., polyethylene glycol, polycaprolactone)	Tunable mechanical properties, degradation rate, and porosity; customizable for specific needs	Polyethylene glycol (PEG): Tailorable mechanical properties. Polycaprolactone (PCL): Provides long-term structural support
Decellularized Matrix	Natural tissues stripped of cellular components	Preserves native ECM structure and biochemical cues; can induce immunogenicity if not properly processed	Cardiac ECM: Mimics native tissue architecture and biochemical composition. Requires thorough decellularization to minimize immunogenicity
Hydrogels	Highly hydrated, water-swollen networks (e.g., alginate, hyaluronic acid)	Mimic ECM environment, allow cell encapsulation, but generally have poor mechanical strength	Alginate: Encapsulates cells for transplantation. Hyaluronic acid: Enhances cell proliferation and migration
Nanofibrous Scaffolds	Nano-sized fibers (e.g., electrospun fibers)	High surface area, mimic ECM architecture, provide mechanical support and promote cell adhesion	Electrospun polycaprolactone (PCL) fibers: Mimic native ECM architecture, provide structural support for cell growth and alignment
Composite Scaffolds	Combination of different materials (e.g., natural/synthetic polymers, ceramics)	Synergistic properties, tailored mechanical and biological characteristics	Collagen-chitosan: Enhanced mechanical strength and cell adhesion. Gelatin-PCL: Mimics native ECM while providing mechanical support

### 3.1 Tissue engineering: 3D printed scaffolds

The kind of scaffolding refers to the specific design or structure used to support workers and materials during construction or maintenance activities. Tissue engineering is a multidisciplinary field that involves the application of engineering principles and techniques to develop functional biological tissues. The utilization of three-dimensional (3D) printing technology in the construction industry has led to the development of 3D printed scaffolding.

For a scaffold to be deemed suitable, it is imperative that it effectively aids in the healing process by facilitating the utilization of natural extracellular matrix (ECM) texture. The utilization of three-dimensional printing in tissue engineering has facilitated the exact manipulation of scaffold production, resulting in the preservation of the natural extracellular matrix (ECM) shape. Scaffolds fabricated with three-dimensional geometric specifications have a finely detailed structure employing a combination of hybrid and heterogeneous materials, therefore emulating the morphological characteristics of natural extracellular matrices (Duan et al., 2011; Dhania et al., 2022; Sun T. et al., 2023). In order to replicate the supplementary capability of the endothelium network, it is possible to introduce channels on the surface of the 3D scaffold. The three-dimensional patterns, grooves, and channels have an impact on cell motility, morphology, phenotypic, and physiology. In the context of stem cells (SCs), the phenomenon referred to as topological guidance or surface is of significant importance in the determination of their many phenotypes, including but not limited to adipose, osteoporotic, neurogenic, myogenic, and cardiomyogenic. Understanding the content and structure of the extracellular matrix (ECM) is of utmost importance in order to effectively regulate cell differentiation and manipulate cell physiology. The replication of these important traits can be achieved through the utilization of computer-aided modeling and the fabrication of 3D printed scaffolds. Thus far, the implementation of narrow channels has been executed with a notable level of precision. Additionally, it has been observed that narrow patterns can potentially impact the process of cell adhesion. A study conducted by researchers observed the concurrent motion of CM-implanted scaffolds within a single day following cell culture. This finding suggests that the attachment of individual cells to the scaffold inside the micro-channels occurred swiftly, and the establishment of channel connection was equally quick (Duchemin et al., 2019; Yang et al., 2024).

In addition, a scaffold based on high-resolution extracellular matrix (ECM) was fabricated by the utilization of the multiphoton-excited 3D printing method. The researchers utilized a three-dimensional scaffold to cultivate a population of five-x104 human myocytes, which were produced from pluripotent stem cells (hiPSC-CM), smooth muscle cells (SMCs), and endothelial cells (EC) in a ratio of 2:1:1:1. The objective of this experiment was to generate human cardiac myocyte patches (hCMP). Within a 24-h period following the initiation of seeding, the presence of human cardiac myocytes (hCMP) was seen, characterized by the expression of cardiac protein markers and the occurrence of transitory calcium signaling, accompanied with concurrent normal ventricular contractions. The scaffold was employed in the treatment of an animal model of myocardial infarction (MI) and served as a means to control for hypertrophic cardiomyopathy (hCMP) in the absence of a scaffold (Fang et al., 2022). When compared to the control group, the utilization of a 3D printed scaffold-based hCMP resulted in notable enhancements in several aspects of cardiac physiology, including infarct size reduction, increased cell survival, higher endothelial density, and enhanced cell proliferation. A new application of a composite scaffold composed of polycaprolactone (PCL) and PCL carbon nanotubes involves its utilization in the field of cardiac tissue engineering, where its biocompatibility has been assessed. For the goals of tissue engineering, PLGA scaffolds were fabricated in a similar manner with the same material (Fazal et al., 2021). The use of cells and biological materials within scaffolds using advanced 3D printing technology enables the synthesis of full or partial organs (Table 2) (Gao L. et al., 2022).

**TABLE 2:** Aspects of scaffolding with applications.

Aspect of scaffolding	Construction industry application	Tissue engineering application
Definition	Design or structure to support workers and materials during construction	Application of engineering principles to develop functional biological tissues
Technological Advancement	Utilization of 3D printing technology for development of 3D printed scaffolds	Utilization of 3D printing for exact manipulation of scaffold production, preserving ECM shape
Structural Characteristics	- Three-dimensional geometric specifications	- Finely detailed structure mimicking morphological characteristics of natural ECM.
Cellular Interaction	- Introduction of channels impacting cell behavior	- Impact on cell motility, morphology, and phenotypic changes
Stem Cell Phenotypes	- Topological guidance for determination of various phenotypes	- Influence on adipose, osteoporotic, neurogenic, myogenic, and cardiomyogenic phenotypes
Replication of ECM Traits	- Utilization of computer-aided modeling for replication	- Replication of ECM traits for effective regulation of cell differentiation and physiology
Precision	- Implementation of narrow channels with high precision	- Fabrication of high-resolution ECM-based scaffolds using multiphoton-excited 3D printing method
Experimental Outcome	- Swift attachment and connection of cells inside micro-channels	- Successful cultivation of human myocytes, resulting in cardiac myocyte patches
Benefits	- Enhanced cell adhesion and proliferation	- Enhanced cardiac physiology, reduced infarct size, increased cell survival and endothelial density
Material Utilization	- Composite scaffold composed of PCL and PCL carbon nanotubes	- Fabrication of PLGA scaffolds for tissue engineering goals
Future Possibilities	- Synthesis of full or partial organs using advanced 3D printing technology	- Synthesis of full or partial organs using biological materials within scaffolds

### 3.2 Microfluidics-based approaches in 3D cardiac tissue engineering

The provision of oxygen and nutrients to thick heart tissue (>200–200 μm) is a substantial obstacle to tissue engineering. In order to effectively treat ischemia disorders, it is necessary to establish an infiltrated microvascular network that closely resembles the typical arterial vascular network. In order to stimulate angiogenesis in a living organism, endothelial cells (EC) were either transplanted or separate organs were re-endothelialized, resulting in the formation of microvascular structures. Nevertheless, it is important to acknowledge that there exist limits to the aforementioned approaches (Gao et al., 2020). In recent times, there has been a notable advancement in the field of microfluidic devices, specifically in the context of showcasing heart functionality on a chip. This has been achieved by the utilization of advanced microvascular tissue engineering techniques (Fazal et al., 2021). Microfluidic devices may be fabricated by employing computer-aided design techniques, as well as employing precise electrical and mechanical control mechanisms for fluid manipulation. Additionally, the use of three-dimensional coating methods enables the incorporation of biological components into these devices. Microfluidic devices have the capability to offer essential characteristics of functioning tissue units at both the microscopic and nanoscale levels, as demonstrated by organ-on-a-chips and lab-on-a-chips (Gerdes et al., 2021). Microfluidic devices may be fabricated by employing computer-aided design techniques, as well as employing electrical and mechanical means to regulate fluid flow. Additionally, the application of 3D coating methods enables the incorporation of biological components into these devices. Microfluidic devices enable the microscopic and nanoscale visualization of essential characteristics of functioning tissue units, as demonstrated by organ-on-a-chips and lab-on-a-chips (Gerdes et al., 2021). The evaluation of cardiotoxicity *via* cardiac magnetic resonance imaging (McMahan et al., 2020) and medication screening. The physiology of cardiac ventricular contractions was investigated by researchers through the utilization of a microfluidic technology. The utilization of flexible fibronectin pattern elastomeric tubes in the cultivation of anisotropic muscle tissue has been successfully employed to fabricate two-dimensional muscle thin films (MTFs) that accurately replicate the anisotropic characteristics observed in the ventricular lining of the heart. The 2D configuration exhibited an elevated degree of both systolic and diastolic state. Cells cultured in a two-dimensional (2D) arrangement and subjected to electrical pressure are capable of generating contractile forces within quantifiable limits. This method was further employed in their drug screening programs. In a similar vein, Gintant et al. (2019) conducted an experiment utilizing a CM stimulus-stimulated micro-stimulus to showcase the generation of an artificial heart rhythm by microfiltration on a microbiological column composed of polymethyloxane (PDMS). A heart pump was developed on a microfluidic device by aligning the cell plates and fluid within microfluidic channels. Sheehy et al. aimed to recreate the physiological functionality of mature cardiac tissue and assess the protein expression inside this tissue. A heart chip *in vitro* model was built (Gonzalez de Torre et al., 2020). The chip was inoculated with complementary metal-oxide semiconductors (CMs). The study revealed that anisotropic engineering of the myocardium exhibits a global alignment, generates contractile stress, and has an inotropic concentration response to the adrenergic agonist isoproterenol (Gorjipour et al., 2021). The synthetic myocardium exhibited a comparable expression pattern of myofibril genes to that observed in muscle fibers isolated from mature rat ventricular tissue. The current advancements in technology have enabled the manipulation and replication of cellular production, environmental conditions, regeneration, and control in real-time. Additionally, the visualization of cellular and morphogenic processes has now become feasible. Bioreactors has the capability to create a harmonious milieu conducive to the optimal biological performance of cardiac cells. Furthermore, these techniques enable effective integration into the process of cardiac tissue remodeling, in contrast to 3D tissue engineering procedures that do not precisely replicate the characteristics of the tissue seen in its natural environment (Table 3) (Guo et al., 2020).

**TABLE 3:** Aspects of microfluidics-based approaches in 3D cardiac tissue engineering.

Aspect					
Synthesis Methods	Hydrogel encapsulation, 3D bioprinting, Self-assembly, Microscale patterning	Precise control over microenvironment, Tailored mechanical properties	Complex fabrication processes, Limited scalability	Tailored mechanical properties, Controlled drug release	Heart-on-a-chip, Cardiac patches, Organs-on-chips
Advantages	High spatial resolution, Precise control over microenvironment, Tailored mechanical properties	Mimics physiological conditions, Real-time monitoring capabilities	Time-consuming optimization, High cost of equipment	Enhanced cell-cell interactions, Controlled flow conditions	Enhanced drug testing and screening capabilities
Disadvantages	Complex fabrication processes, High cost of equipment, Time-consuming optimization	Reduced need for animal testing, Reduced risk of contamination	Limited scalability, Potential for crosstalk between channels	Integration with sensing and imaging technologies	Limited scalability, Potential for crosstalk, Reduced scalability for industrial production
Properties	Tailored mechanical properties, High cell viability, Controlled drug release	Controlled flow conditions, Mimicking physiological conditions	High spatial resolution, Enhanced cell-cell interactions	Tailored drug release kinetics, Enhanced cell signaling	Mimicking physiological conditions, Enhanced cell attachment and alignment
Examples in Heart	Heart-on-a-chip, Cardiac patches, Organs-on-chips	Controlled drug release, Enhanced cell signaling	Heart-on-a-chip, Cardiac patches, Organs-on-chips	Organs-on-chips, Engineered heart tissue	Engineered heart tissue, Organs-on-chips, Heart valve models

## 4 Advanced techniques in cardiac tissue engineering

Advanced methods in cardiac tissue engineering encompass 3D bioprinting, heart-on-a-chip (HoC) technology, and the creation of tissue-engineered cardiac scaffolds. 3D bioprinting allows for the sequential placement of biomaterials to create cardiac constructions that closely resemble the form and function of natural hearts (Bejleri et al., 2018; Wang et al., 2018; Chingale et al., 2022). HoC technology integrates cardiac tissue engineering with microfluidics to replicate human heart physiology and medication reactions in three-dimensional models. In addition, tissue-engineered cardiac scaffolds strive to mimic the mechanical properties of natural myocardial tissues by employing synthetic materials like hydrogels to accomplish certain features such as non-linear elasticity, anisotropy, and viscoelasticity. These sophisticated methods tackle difficulties in the field of cardiac tissue engineering, providing inventive remedies for regenerative medicine and study on cardiac diseases (Table 4) (Lu et al., 2023).

**TABLE 4:** Most common technique in Cardiac Tissue Engineering.

Technique	Advantages	Applications in heart	Disadvantages	Reference
Biomimetic Scaffolds	Mimics natural extracellular matrix	Cardiac tissue regeneration	Lack of long-term stability	Bertsch et al. (2023)
3D Bioprinting	Precise control over tissue structure	Customized tissue patches, vascularization	Limited scalability	Kuru et al. (2024)
Decellularization	Preserves native tissue architecture	Scaffold for cell seeding, reduced immunogenicity	Loss of bioactive molecules	Golebiowska et al. (2024)
Stem Cell Therapy	Potential for regeneration	Cardiomyocyte replacement, angiogenesis	Risk of tumorigenesis	Abubakar et al. (2023)
Gene Editing	Targeted modification of genetic defects	Correction of inherited cardiac disorders	Off-target effects	Zielińska et al. (2023)
Nanotechnology	Enhanced drug delivery	Targeted therapy for cardiac diseases	Potential toxicity	Alzoubi et al. (2023)
Electrospinning	High surface area for cell attachment	Engineering cardiac patches, improved integration	Limited mechanical strength	Gomes et al. (2022)
Microfluidics	Precise control over microenvironment	Drug screening, mimicking blood flow *in vitro*	Complexity in scaling up	Abolhassani et al. (2024)
Tissue Engineering	Tailored mechanical properties	Replacement of damaged myocardium, myocardial repair	Integration with host tissue	Sajjad et al. (2024)
Synthetic Biology	Programmable cellular behavior	Enhanced tissue functionality, disease modeling	Ethical concerns	Bohua et al. (2023)
Immunomodulation	Modulates immune response	Reduced rejection, improved tissue integration	Potential for immunosuppression	Zhong et al. (2023)

### 4.1 Bioreactors and hybrid systems for cardiac tissue engineering

Due to the implementation of the hybrid bioreactor methodology, researchers have successfully utilized microfluidic systems to facilitate the provision of nutrients and the exchange of metabolites and gases. These processes pose challenges when cells undergo proliferation in layers that are several millimeters thick (Aksu et al., 2023). Previous research has demonstrated that microfluidization has the potential to enhance cell viability in cardiac tissues with thicknesses measuring in the range of several millimeters. Guo et al. (2021) employed a microcyte perfusion system to provide oxygen to the seed cells, resulting in the development of thicker and more efficient cardiac tissue. The investigators presented a scaffold utilizing a 3D bioreactor, which had many parallel channels designed to mimic a capillary network. This configuration was intended to meet the nutritional requirements of the cells and improve cellular perfusion (Aksu et al., 2023). The cell culture medium employed a perfluorocarbon oxygen compound (PFC) as a substitute for hemoglobin, the natural oxygen carrier molecule, in order to meet the oxygen needs of the seed cells. Utilizing the aforementioned model, the researchers conducted calculations pertaining to oxygen consumption and examined the correlation between tissue oxygen distribution and several factors such as the physical dimensions of the canal, flow rate, PFC concentration, and cell density (Jana et al., 2021). This model serves as a foundation for studying tissue engineering with specified thicknesses and densities, as well as for replicating the process of heart muscle tissue creation within a realistic setting. Micro-channel perfusion bioreactor scaffolds are employed in the field of cardiac tissue engineering to induce stimulation in heart tissue. The regulation of hydrodynamic shear stress in the cardiac tissue structure is achieved by means of medium currents in micro-channels and weld coupling, leading to improved physiological outcomes (Karbassi et al., 2020). In this study, the author assesses the cultivation of CM behaviors in microchannels by identifying low thresholds for elevated contractile domains, increased recordings indicative of enhanced intracellular connectivity, contractions, and cellular electrical excitability (Kawai et al., 2021).

### 4.2 Scaffold-free 3D bioprinting for cardiac tissue engineering

**FIGURE 4:**
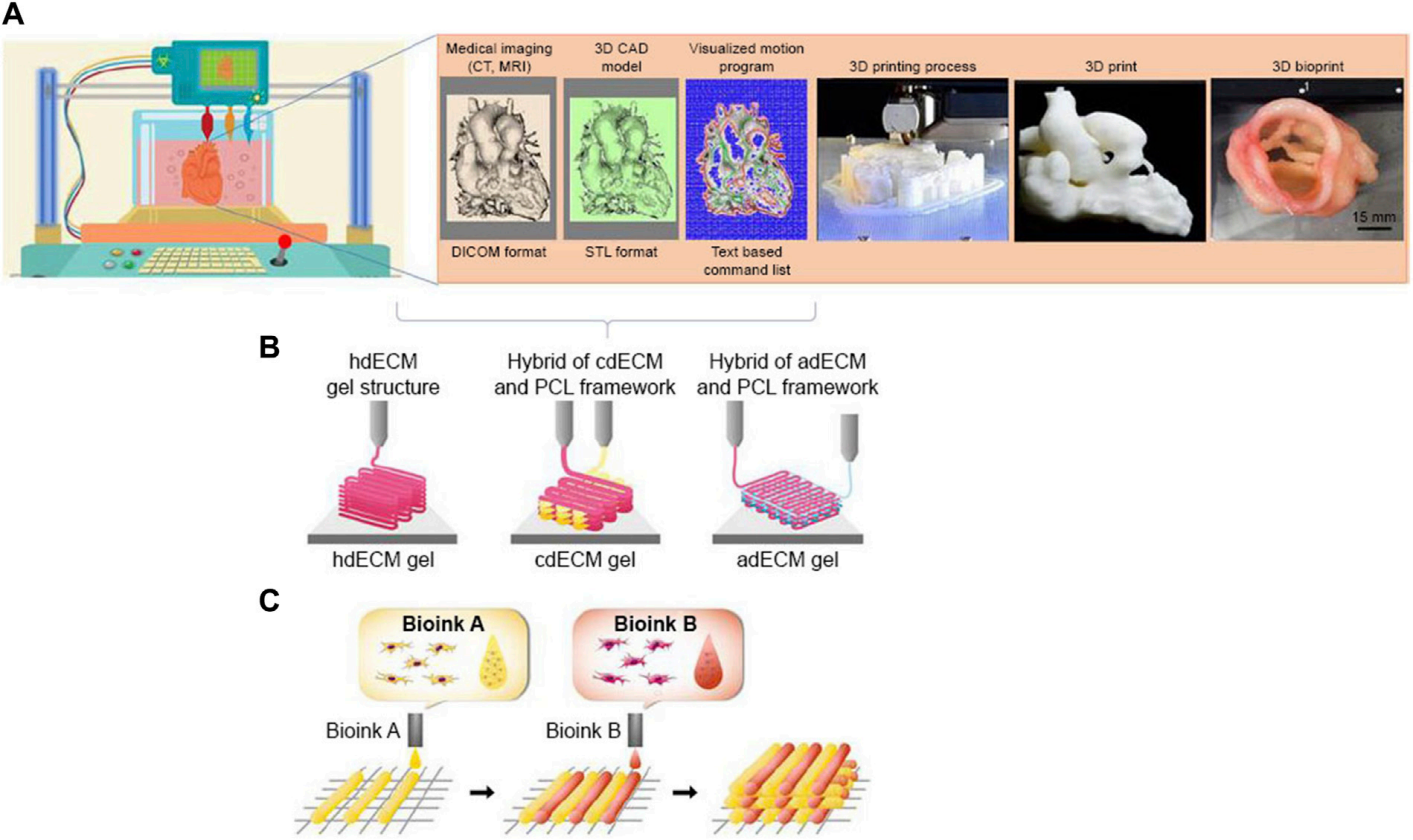
Process of 3D bioprinting, **(A)** steps of 3D bioprinting, **(B)** pre-scaffold fabrication bioprinting, **(C)** simultaneous hybrid 3D bioprinting (Qasim et al., 2019)

Despite the effectiveness of scaffolding in tissue engineering, the use of these materials still poses some problems, including immune response and degradability. In certain instances, the utilization of scaffolding may lead to an adverse autoimmune response, resulting in potential harm or injury to the affected region. In severe circumstances, this reaction might even pose a threat to an individual’s life (Kawai et al., 2021). Artificial structures can be built by the implementation of 3D scaffolding or in the absence of scaffolding. Bio-3D printing employs cell adherence as a means to compensate for the absence of scaffold support. Bioinks are a crucial component of 3D bioprinting technology for cardiac tissue engineering and regeneration the purpose of utilizing bioinks is to fabricate three-dimensional bio-scaffolds that emulate the cardiac milieu and furnish a conducive framework for cellular proliferation and differentiation (Figure 4) (Liu et al., 2022). There are two distinct classifications of bioinks based on their source: natural and synthetic. Natural bioinks are characterized by a higher degree of biocompatibility and biodegradability, whereas synthetic bioinks provide enhanced manipulation of scaffold properties. Bioinks for cardiac tissue engineering must exhibit robust material qualities, given the circulatory system’s demand for strength, flexibility, and durability. The function of bioinks is essential in facilitating the delivery of oxygen and nutrients to the developing tissue, as well as the removal of metabolic wastes (Ghosh and Yi, 2022). Additionally, they aid in the assimilation of the designed tissue with the vascular system of the host. Biomaterials, including collagen, gelatin, and alginate, are frequently included in the formulation of bioinks for cardiovascular purposes (Benwood et al., 2021). These biomaterials possess the potential to surpass the constraints of existing therapies for coronary heart disease. The optimal characteristics of bioink: The optimum bioink utilized in cardiac tissue engineering should possess the ability to support the development of mature contractile phenotypes in cardiomyocytes and enable effective intercellular communication with neighboring cells (Wang et al., 2021). Recent breakthroughs in the field of 3D printing and bioprinting have facilitated the creation of bio fabricated scaffolds through the utilization of biomaterials and bioinks. These scaffolds are characterized by their high porosity and intricate architectures, making them suitable for applications in cardiac tissue engineering. The achievement of three-dimensional bioprinting can be facilitated by many techniques, including pellet culture, drop-drop, hydrodynamic cell entrapment, rotating flasks, liquid cladding, micro-molding, and ship rotation (Khanna et al., 2022). The phenomenon of cell adhesion serves as a catalyst for the prompt cellular differentiation within three-dimensional (3D) settings. To achieve a tissue-like structure, cells may be layered using 3D printer scaffolding and assembled with other cells. In a recent research (Kheila et al., 2021), scaffold-free structures were fabricated utilizing micro alginate droplets. The formation of micro alginate droplets on the substrate layer was achieved by determining the thickness of the layer and using ring hydrogel molds. These droplets were then seeded with a mixture of EC and smooth muscle cells in a 1:1 ratio (Kheila et al., 2021). The phenomenon seen was a succession of individual cells. Additionally, the utilization of scaffolds or compounds in biological inks might result in degradability, immunological responses, and toxicity. The utilization of 3D printing technology for the delivery of stem cells, without the incorporation of biological substances, circumvents the occurrence of these difficulties. Spherical cellular structures were generated by employing a mixture of human induced pluripotent stem cell-derived cardiomyocytes (hiPSC-CM), fibroblasts (FB), and endothelial cells (EC) in varying proportions of 70:15:15, 70:0:30, and 45:40:15. The process of fabricating cardiac patches using three-dimensional printing was conducted utilizing a set of nine spherical objects. According to the cited source (Kikuchi et al., 2022), during the initial stage of needle arrangement, the patches exhibited spontaneous beating for a duration of 3 days. Subsequently, over the next 2 days, the gaps between the patches underwent fusion. During the process of patching, the alignment of ventricular and uniform cells was noted, along with the assessment of electrical conductivity. Furthermore, the extracellular matrix (ECM) not only facilitates cell-cell contact and electromechanical connection, but also enhances the natural composition of the tissue by minimizing the presence of superfluous elements inside these three-dimensional structures. Tissue-spheroid-based organ bioprinting is widely recognized as the prevailing approach in the field of 3D printing. The arrangement of cells in a stacked configuration results in the formation of a highly structured cellular aggregate. There exists a potential possibility to develop completely operational cardiac organs or substantial tissues for the purpose of treating myocardial infarction (Kim et al., 2021).

Esser et al. (2023) introduced a novel method for 3D-bioprinting human induced pluripotent stem cell (hiPSC)-derived cardiomyocytes within a collagen–hyaluronic acid ink. This approach enabled the creation of large functional cardiac tissues in ring and ventricle shapes with precise and consistent results. These printed tissues showcased organized sarcomeres in the hiPSC-derived cardiomyocytes, demonstrating spontaneous and rhythmic contractions that persisted for extended periods. Importantly, the tissues exhibited the ability to contract effectively even when faced with passive resistance. In their study, Roshanbinfar et al. (2024) developed an injectable hydrogel made of collagen and PEDOT:PSS, which effectively shields infarcted hearts from ventricular tachycardia (VT). This innovative hydrogel can also be combined with hiPSC-cardiomyocytes to support partial remuscularization of the heart. The incorporation of PEDOT:PSS in the collagen gel enhances gel formation, micromorphology, and conductivity. Cardiomyocytes derived from hiPSCs and embedded in collagen-PEDOT:PSS hydrogels exhibit characteristics resembling adult cardiomyocytes, including near-adult sarcomeric length, improved contractility, enhanced calcium handling, and increased conduction velocity. RNA-sequencing data further confirm the advanced maturation and enhanced cell-matrix interactions within these constructs. Injection of collagen-PEDOT:PSS hydrogels into infarcted mouse hearts effectively reduces VT incidence to levels comparable to healthy hearts. Overall, the collagen-PEDOT:PSS hydrogels provide a versatile platform for addressing cardiac injuries.

In the field of heart tissue engineering, one of the key challenges is the low resolution and low complexity of current 3D-printed heart models (Lu et al., 2023; Kieda et al., 2024). This limitation hinders the ability to accurately replicate the intricate structures and functions of native heart tissue, thereby impacting the effectiveness of these models for research and therapeutic applications (Kato et al., 2021). The low resolution of current 3D-printed heart models refers to the limited level of detail and precision in reproducing the complex architecture of the heart tissue (Giannopoulos et al., 2016). This can result in inaccuracies in the representation of tissue morphology, cell organization, and vascular networks, which are crucial for mimicking the physiological behavior of the heart (Giannopoulos et al., 2016). As a result, the predictive capabilities of these models for studying disease mechanisms, drug responses, and tissue regeneration are compromised. Moreover, the low complexity of current 3D-printed heart models refers to the simplified nature of the structures and functions that can be achieved with existing printing technologies (Jain et al., 2022). While some progress has been made in creating basic cardiac constructs, such as myocardial patches or simple tissue scaffolds, the ability to replicate the full complexity of the heart, including its multi-scale organization, heterogeneous cell populations, and dynamic mechanical properties, remains a significant challenge (Akbarzadeh et al., 2023). Addressing these limitations in 3D-bioprinting for heart tissue engineering requires advancements in printing technologies, biomaterials, and tissue engineering strategies. Researchers are actively exploring new approaches, such as high-resolution printing techniques, bioinks with improved bioactivity and mechanical properties, and innovative scaffold designs that can better recapitulate the native heart tissue architecture. By overcoming these challenges, the field can progress towards developing more physiologically relevant and clinically translatable 3D-printed heart models for research, drug screening, and regenerative medicine applications (Saini et al., 2021).

## 5 Materials selection for heart tissue engineering scaffolds

In the realm of heart tissue engineering scaffolds, the selection of materials is crucial for successful regeneration. Various materials have been explored, including synthetic biodegradable macromolecules like aliphatic polyesters, polyurethane, and poly (glycerol sebacate). Additionally, the incorporation of oxygen-generating microparticles within hydrophobic polymers like polycaprolactone has shown controlled oxygen release kinetics, enhancing cell viability and proliferation (Mohammadi Nasr et al., 2020). Chitosan scaffolds have also been highlighted for their biocompatibility, porosity, and biodegradability, mimicking the heart’s extracellular matrix. Furthermore, electrospun nanofibers derived from heart extracellular matrix, in combination with PVP, gelatin, and polycaprolactone, have demonstrated promising characteristics for cardiac tissue engineering, offering biomimetic scaffolds with suitable surface topography and mechanical properties. These diverse materials present valuable options for constructing functional and effective scaffolds in cardiac tissue engineering (Table 5) (Biglari and Zare, 2024).

**TABLE 5:** An extensive review on materials commonly used in heart tissue engineering scaffolds.

Material	Biocompatibility	Bioresorbability	Mechanical properties	Porous structure	Cell adhesion and growth	Degradation rate	Tunable properties
Collagen	High	Yes	Low to moderate	Yes	Excellent	Moderate	Limited
Alginate	High	Yes	Low	Yes	Moderate	High	Yes
PLGA	High	Yes	High	Yes	Moderate	High	Yes
Fibrin	High	Yes	Low to moderate	Yes	High	Moderate	Limited
Decellularized ECM	High	Yes	Variable	Yes	High	Variable	Limited
Polyethylene Glycol	High	No	Low to high	Yes	Low	Slow	Yes
Gelatin	High	Yes	Low to moderate	Yes	Moderate	Moderate	Limited
Silk	High	Yes	Moderate	Yes	Moderate	Moderate	Yes
Hyaluronic Acid	High	Yes	Low to moderate	Yes	Moderate	Moderate	Yes
Chitosan	High	Yes	Low to moderate	Yes	Moderate	Moderate	Yes
Hydrogel	High	Yes	Low to moderate	Yes	Moderate	Variable	Yes
Nanogels	High	Yes	Low to moderate	Yes	High	Variable	Yes
PCL (Polycaprolactone)	High	Yes	Moderate to high	Yes	Moderate	Slow	Yes
PGA (Polyglycolic Acid)	High	Yes	High	Yes	Moderate	Fast	Yes
PVA (Polyvinyl Alcohol)	High	Yes	Low to moderate	Yes	Moderate	Moderate	Yes
PMMA (Polymethyl Methacrylate)	Moderate	No	High	No	Low	Slow	Yes
HA-PLGA (Hyaluronic Acid-PLGA)	High	Yes	Moderate	Yes	High	Moderate	Yes
PEGDA (Polyethylene Glycol Diacrylate)	High	Yes	Low to moderate	Yes	High	Moderate	Yes
Matrigel	High	Yes	Low to moderate	Yes	High	Variable	Limited
Collagen-GAGs (Glycosaminoglycans)	High	Yes	Low to moderate	Yes	High	Moderate	Yes
PLCL (Poly(L-lactide-co-ε-caprolactone))	High	Yes	Moderate	Yes	Moderate	Moderate	Yes
PPF (Poly(propylene fumarate))	High	Yes	Moderate to high	Yes	Moderate	Moderate	Yes
PEGT/PBT (Poly(ethylene glycol)-co-(poly(butylene terephthalate)))	High	Yes	Low to moderate	Yes	Moderate	Moderate	Yes
PEG-PCL (Poly(ethylene glycol)-poly(ε-caprolactone))	High	Yes	Moderate	Yes	High	Moderate	Yes
PCL-PEG-PCL (Poly(ε-caprolactone)-poly(ethylene glycol)-poly(ε-caprolactone))	High	Yes	Moderate	Yes	High	Moderate	Yes
PEG-PLGA (Poly(ethylene glycol)-poly(lactic-co-glycolic acid))	High	Yes	Low to moderate	Yes	High	Moderate	Yes
Collagen-PLGA (Collagen-poly(lactic-co-glycolic acid))	High	Yes	Low to moderate	Yes	High	Moderate	Yes

Current issues with scaffolds for heart tissue engineering include problems related to loading, steric hindrance, and cell positioning (Rajab et al., 2020; Jalilinejad et al., 2023). One of the challenges in scaffold design for heart tissue engineering is achieving proper loading conditions. The scaffold needs to be able to withstand the mechanical forces exerted by the beating heart, while also providing the necessary support for cell growth and function. Inadequate loading conditions can lead to scaffold failure, poor tissue formation, or decreased functionality of the engineered tissue (Roacho-Pérez et al., 2022; Baghersad et al., 2023). Another issue with scaffolds is steric hindrance, which refers to the interference of scaffold materials with cell growth and communication. The scaffold’s architecture and composition must be carefully designed to allow for proper cell infiltration, nutrient exchange, and waste removal. If the scaffold material is too dense or obstructive, it can impede cell migration and tissue formation, resulting in suboptimal tissue engineering outcomes (Majid et al., 2020b; Boroumand et al., 2021). Proper cell positioning within the scaffold is critical for the development of functional heart tissue. Cells need to be organized in a specific spatial arrangement to mimic the native structure of the heart and promote proper tissue function. However, achieving precise cell positioning within the scaffold can be challenging, especially in larger tissue constructs. Without proper cell alignment and organization, the engineered tissue may lack the necessary functionality and integration with the host tissue (Dattola et al., 2019; Burnstine-Townley et al., 2020). To address these challenges, researchers are exploring innovative scaffold materials and fabrication techniques that can enhance loading capabilities, reduce steric hindrance, and improve cell positioning. For example, the development of biomimetic scaffolds that mimic the extracellular matrix of the heart can promote cell adhesion, migration, and differentiation (Zhang et al., 2023). Additionally, advanced 3D printing technologies allow for the precise control of scaffold architecture and cell distribution, enabling more accurate cell positioning within the scaffold (Iturriaga et al., 2021). By overcoming these challenges, researchers can advance the field of heart tissue engineering and develop more effective strategies for repairing damaged heart tissue.

### 5.1 Exploring biological materials and their role in scaffold design

The scaffold functions as the primary substrate for enhancing and generating tissue. The molecular, functional, and mechanical characteristics of scaffolds play a crucial role in facilitating the viability and specialization of cells (Kulvinskiene et al., 2020). To facilitate the development of a scaffold capable of providing structural support to cells, it is imperative for biological materials to contain the following characteristics (Lakshmanan and Maulik, 2018). To achieve a high degree of resemblance to the host tissue, the aforementioned characteristics are applicable across various engineered tissue types. Biomaterials and scaffolds utilized in the context of artificial cardiovascular and bioprotein correction necessitate a diverse array of characteristics, including degradability, biocompatibility, flexibility, durability, and immunogenicity. The scaffolds exhibit diverse dimensions and possess the ability to be customized for cardiac applications, owing to the wide range of cardiac abnormalities and patient-specific factors (Litowczenko et al., 2021). The induction of vascular neovascularization by this biological component is seen advantageous as it facilitates the sufficient oxygenation of the tissue. The proposed outcome of this approach is the formation of little scar tissue and the absence of thrombotic risk. The presence of the latter material may need the administration of anticoagulants (Liu et al., 2018). In addition, it is imperative for these biomass scaffolds to possess bioactivity, facilitating cellulose promotion both *in vitro* and *in vivo*, while concurrently enhancing cell efficiency and exhibiting rapid degradation (Malandraki-Miller et al., 2018). The utilization of tissue engineering (TE) as a traditional therapeutic approach is limited by the accessibility and cost-effectiveness of biological materials and scaffolds (Mancuso et al., 2020). The utilization of natural and synthetic polymers is prevalent in cardiovascular tissue engineering since they serve as the primary biological components. Among the several techniques employed for scaffold construction, the electrical approach has been identified as the most effective (Marchini and Gelain, 2022).

### 5.2 Diversity of polymers used in cardiac scaffold fabrication

Different polymers are used in the production of cardiac scaffolds to tackle the difficulties in cardiac tissue engineering. Alginate, chitosan, collagen, fibrin, gelatin, and glycosaminoglycan are often used natural polymers because they are biocompatible and can facilitate cell adhesion, proliferation, and differentiation (Mohammadi Nasr et al., 2020). Aliphatic polyesters, polyurethane, and poly (glycerol sebacate) are examples of synthetic biodegradable macromolecules that are highly valued for their mechanical qualities and ability to work well with heart tissue. Furthermore, innovative composite scaffolds that include gelatin, chitosan, and functionalized multi-walled carbon nanotubes have demonstrated potential in the field of cardiac tissue engineering. These scaffolds offer improved electrical conductivity and mechanical properties. These various polymers are essential in creating scaffolds that possess the required attributes for the regeneration of the heart and the creation of functional tissue (Godinho et al., 2022).

## 6 Synthetic polymers in cardiac scaffold design

Possessing the appropriate information may facilitate the efficient production of materials. The development of synthetic polymers was motivated by the necessity to rebuild cardiac tissue using appropriate biological materials (Mayorca-Guiliani et al., 2019). Synthetic polymers have the advantageous characteristic of being readily manufacturable and manipulable. The physical qualities of a polymer may be utilized to control its mechanical properties, molecular weight, heterogeneity index, and degradation rate (Fazal et al., 2021). Sutures and meshes are frequently fabricated using synthetic polymers due to their biocompatibility and superior mechanical characteristics (Kheila et al., 2021). Polyglycolic acid (PGA) and polylactic acid (Hu et al., 2023) are commonly employed in the field of cardiac surgery. These two polymers have been employed either individually or in a 50:50 ratio as a biomaterial for tissue regeneration in pediatric patients diagnosed with congenital heart disease (McMahan et al., 2020). In 1999, Carrier conducted a study that showcased enhanced ultrastructure and metabolic activity within rotary bioreactors by the utilization of PGA scaffolds. Rotational bioreactors have been observed to exhibit a decrease in both cell adhesion and cell damage (Mohammadi Amirabad et al., 2017). According to a study conducted by researchers (Nazari et al., 2021), it has been shown that the utilization of rotational bioreactors leads to an enhancement in cell adhesion while simultaneously reducing cell damage. The primary worry lies in the toxicity associated with synthetic polymers. Consequently, there is a growing consideration for the utilization of poly-L-lactic acid (PLLA). Vascular tissue engineering (TE) has demonstrated promising outcomes with the utilization of poly-L-lactic acid (PLLA) and mesenchymal stem cells produced from bone marrow (BM-MSC). In a study conducted by researchers, the efficacy of nanofiber scaffolds composed of PLLA was established by their implantation into live animal models, specifically rats. The findings revealed that these scaffolds possess the ability to be reconstituted with cellular and extracellular matrix (ECM) components, including native artery material (Fang et al., 2022). As per Hashi’s findings, nanofiber scaffolds composed of PLLA has the capability to undergo reconstitution with cellular and extracellular matrix (ECM) components, therefore incorporating native artery content. In this study, both cells and scaffolds were surgically transplanted into the common carotid artery of rats. In contrast to cell transplants, scaffolds that were seeded with mesenchymal stem cells (MSC) demonstrated minimal platelet aggregation at the surfaces of the lumens (Gerdes et al., 2021). According to the MSC (MSC), (Lakshmanan and Maulik, 2018). In this study, both cellular components and cell scaffolds were surgically inserted into the common carotid artery of live animal models, namely, rats (Naureen et al., 2021). The cell scaffolds were inoculated with mesenchymal stem cells (MSC) and shown significantly reduced levels of platelet aggregation at the inner surfaces of the luminal region, as compared to cell transplants. The reason for this occurrence might be attributed to the anti-tightness characteristic of MSC (Gonzalez de Torre et al., 2020). Poly-L-lactic acid (PLLA), when undergoing degradation within the human body, has the potential to be eliminated by the excretion of carbon dioxide and water. Polyurethane has biocompatibility, yet it lacks biodegradability until it undergoes copolymerization. Polyurethane, when used in conjunction with other materials such as siloxane films, cellulose, urea, and PLLA, has been explored for its potential in cardiac tissue repair (Gonzalez de Torre et al., 2020). The utilization of Poly (ε-caprolactone) in conjunction with other biological materials has demonstrated notable efficacy as a composite for the purpose of cardiac tissue healing. They have been employed in conjunction with PLLA only, PLLA and collagen, polypyrrole and gelatin, polyglycolic acid, poly (hydroxymethylglycolide), chitosan and gelatin. According to Nazari et al. (2021), the authors have utilized electro-synthesis of Polyvinylidene fluoride-tetrafluoroethylene (PVDF-TrFE) to fabricate a piezoelectric scaffold with the aim of cardiac tissue engineering. There has been a proposition positing that the amalgamation of natural polymers with synthetic polymers might potentially enhance cell adhesion. There have also been discussions on the utilization of unadulterated natural polymers for polymer applications (Khanna et al., 2022). The table provided below (Table 6) offers a comprehensive explanation of Polymeric Materials for Cardiac Patch.

**TABLE 6:** Other polymeric materials for cardiac patch.

Stem cell	Pathway signaling	Polymer	Porosity/pore size	Mean deformation in an elastic body (MPa) compressive stress excessive tension (MPa) the yield stress of a tensile material	*In vivo* model	Ref
Cardiomyocytes differentiated from hESC	**_**	Poly (glycerol sebacate)	**_**	**_**	Adult male Sprague Daw ley rats	Gomes et al. (2022)
cSca-1, BMMCs, and ADSCs	Anti-vWF), anti-SMA, anti-α-sarcomeric actinin anti-CD31, VEGF, bFGF, and PDGF-bb	Nanopeptides (cell–PuraMatrix™)	**_**	**_**	Wild mice (C57Bl/6J)	Duchemin et al. (2019)
Neonatal cardiomyocytes	Cardiac-specific marker proteins α-actinin, Troponin, β-MHC, and cx43	PGS/collagen	**_**	2.06 MPa (TS) 57.87% (TSA) 83.65% Spitsberg (2005) 4.24 (EM)	**_**	Sayed et al. (2019)
BM-MSCs	CD34, CD45, CD90, CD73, CX43, and cTn T	PPC, PU, and [P (3HB-co-4HB)]	**_**	**_**	**_**	Mancuso et al. (2020)
BM-MSC	CD31, cTnT, and Cx43	PCL/Gelatin	83.6% ± 0.8%/0.83 ± 0.15 μm	**_**	Female Sprague Dawley rats	Gintant et al. (2019)
Cardiac progenitor cells	sca-1, CD34, GATA-4, CD44, CD29, and CD31	PCL/CNT	**_**	11 MPa (EM) 1.3 MPa (TS), 131% Spitsberg (2005)	**_**	Cheema et al. (2012)
BMMSCs	α-sarcomeric actinin, troponin T, CD68, and CD31	PGS/Fibrinogen	**_**	**_**	Farm pigs (*Sus scrofa*) Yorkshire Swine	Liu et al. (2018)
Neonatal rat cardiac cell	SMA +, CD31, Type I collagen and Type IV collagen	Fibrin	**_**	86.0 ± 3.8 (EM) 75.7 ± 11.5 Fazal et al. (2021)	Female Fisher F344 syngeneic immune-competent rats	Gao et al. (2020)
hMSCs	anti-vWF, anti-BRDU	PEG/Alginate	**_**	**_**	Male nude rats (Crl:NIH-Foxn1rnu)	Rhee and Wu (2013)

Numerous natural polymers, including as collagen, gelatin, alginate, silk, fibrin, chitosan, and hyaluronic acid, have been used in the context of heart repair. Natural polymers encompass complex natural constituents that form biodegradable, biocompatible, and readily manipulable matrices. Due to their biocompatibility, ability to enhance cell attachment, and inherent biodegradability without the need for extra treatment or modification, these materials have great potential as suitable candidates for tissue engineering of the heart, despite their suboptimal mechanical characteristics (Rosellini et al., 2018). Collagen stands as the preeminent naturally occurring polymer within the realm of extracellular matrix (ECM) proteins. The tasks of this entity encompass the regulation of biological processes, the formation of structural scaffolds, and the provision of tensile strength (Roshandel and Dorkoosh, 2021). Various studies have described the utilization of different forms of collagen and its alterations for the purpose of cardiac tissue repair (Salem et al., 2022). Fibrin has the potential to serve as a precursor for the fabrication of hydrogels, gels, and microbial structures (Sayed et al., 2019). In addition, growth factors and biological molecules can be included (Bhatt et al., 2022). The regenerative qualities inherent in fibrin glue render it suitable for autonomous use in the repair of heart tissue (Nazari et al., 2021). Following the favorable outcomes observed in the utilization of fibrin patches combined with human embryonic stem cells (hESC-CPC) in the preclinical priming model, hESC-CPC have been employed for the initial instance in the restoration of cardiac stem cells (SCs). In cases of advanced heart failure, characterized by a convincing patient with a functional result of 61. Furthermore, apart from the intramuscular administration of fibrin, several additional investigations have demonstrated its effectiveness as a sealant subsequent to the implantation of stem cells, the development of neural tube structures, or the introduction of liquid mesenchymal cells. Myocardial tissue repair has been proposed as a potential therapeutic approach. Additionally, tissue engineering techniques have been explored for the development of aortic valves. Previous studies have demonstrated in the existing body of literature that chitosan had the potential to serve as a viable biological material for cardiac remodeling (Kulvinskiene et al., 2020). It is widely acknowledged among researchers that the combination of chitosan with other stem cells enhances the integration of stem cells into cardiac tissue (Shafiq et al., 2018). Previous studies have demonstrated a notable improvement in myocardial infarction models just by the utilization of alginate. Moreover, previous studies have demonstrated that the utilization of seeded alginate in combination with stem cells (SCs) has exhibited enhanced efficacy in the restoration of cardiac tissue (Shao et al., 2022; Ge et al., 2023; Zhang et al., 2024). Numerous literature sources have documented the efficacy of Hyaluronic acid in cardiac tissue healing, attributing this effectiveness to its specific molecular weight. The effectiveness of this substance is heavily influenced by its molecular weight, which is 65. The effective transplantation, vascularization, and establishment of linkages with recipient heart cells have been demonstrated in adult rat hearts by the utilization of a subcutaneous gelatin scaffold and/or induction of myocardial infarction. According to previous studies, gelatin has demonstrated the ability to sustain rat cardiomyocyte tissue for a duration of 3 weeks in an *in vitro* setting. This study provides evidence in favor of the advancement of human somatic cell-derived pluripotent stem cells (iPSCs) (Liu et al., 2018). The hydrogel was prepared using autologous stem cells (SC) and fibroblast stem cells (SC) growth factors, namely, basic fibroblast growth factor (bFGF), to provide the controlled release of bFGF for the therapeutic intervention of ischemic cardiomyopathy (Sicari et al., 2023). It has been demonstrated that seeds possess cellular structures. In the study, a canine model was utilized to effectively rebuild a significant defect in the pulmonary artery using collagen thrombin as a triggering agent. Specifically, a collagen thrombin-based fibrinogen/thrombin combination known as Tach Combo was employed (Simon-Yarza et al., 2017). Consequently, this biological material exhibits the potential to be utilized in the reconstruction of low-pressure pulmonary arteries in the context of cardiac surgery, with the aim of restoring the totality of the anomalous lung or substituting for substantial vessels. However, it should be noted that not all of these polymers possess the capacity to effectively control cardiac tissue engineering, and the issue of inflammation persists (Tadevosyan et al., 2021). The table provided below (Table 7) presents an explanation of several natural polymers utilized in the context of cardiac patch applications.

**TABLE 7:** Natural polymers for cardiac patch.

Material	Pathway signaling	Stem cells	Porosity and/or pore size	*In vivo* model	Reference
Collagen	α-SMA	hMSCs	400–600 μm	Male CDF rats	Zarei et al. (2021)
	Anti-vWF and Anti-sarcomeric actin antibody	Autologous SCs	_	Male C57/BL6 mouse	Kikuchi et al. (2022)
	CD105, CD73, Angiogenin, PDGF-B, VEGF, and CXCL1	hMSCs hESC-MC	_	Male athymic RNU nude rats	Shin et al. (2022)
	CD44^+^, CD90^+^, CD45^−^, CD34^−^, α-smooth muscle actinin, Desmine	Autologous mesenchymal SCs	_	Wistar rats	Simon-Yarza et al. (2017)
	Tbx5, Nkx 2.5	hESC-derived cardiovascular progenitors Autologous r-ADSCs	_	Rats of the Wistar strain that are females Rats, both sexes, nuded by Rowett	Mohammadi Amirabad et al. (2017)
Natural Extra cellular matrix	α-SMA, bFGF, vWF, PDGF-B, IGF-1, SMMHC, CD68, and IL-6 HGF, MEF2D, MYH6, Type I collagen	BM-MSCs	91.2% ± 1.3%; 130.5 ± 25.3 mm	Syngeneic male Rats of Lewis	Sicari et al. (2023)
	Cardiotin, Subunit of the Cav 1.2, and cardiac troponin-T Sarcomeric α-actinin, Atrial natriuretic peptide	hMSCs	_	Breeds of adult mutts	Litowczenko et al. (2021), Wang et al. (2022)
	α-MHC, Troponin T, Troponin C, GATA-4, nkx 2.5, α-SMA, Smooth muscle 22α, Fibroblast-specific protein 1, vWF, tie2	Cardiac progenitor cells	_	_	Bianconi et al. (2018)
	Sarcomeric α-actinin, Myosin heavy chain Cardiac troponin T, and vWF	BMMCs	19.5 ± 17.9 μm	_	Malandraki-Miller et al. (2018)

### 6.1 Understanding the role of synthetic polymers in cardiac tissue engineering

In cardiac tissue engineering, synthetic polymers are vital because they provide scaffolds with characteristics that are necessary to imitate heart tissue. Biocompatible, electrically conductive, mechanically strong, and with the right surface properties for cell adhesion and growth—these are the properties of materials made from these polymers. Researchers have looked into using hydrogels, soft polymers, and silicone, among other synthetic polymers, to create artificial heart tissues with mechanical characteristics that are very similar to those of natural cardiac tissue (Suamte et al., 2023). Furthermore, scaffolds made of electroconductive and piezoelectric polymers have demonstrated potential in stimulating cardiomyocytes electrically, which in turn improves electromechanical coupling in cardiac tissues and speeds up their maturation. In order to create trustworthy *in vitro* models for investigating heart disorders and to create living cardiac patches for transplantation, these developments in scaffolds based on synthetic polymers are essential (Wu et al., 2023).

### 6.2 Synthetic and natural polymers for the manufacture of heart stents

Heart stents, pivotal in addressing narrowed or weakened arteries, depend on a diverse array of synthetic and natural polymers for their fabrication, a process that intricately weaves together material science with biomedical engineering principles (Udriste et al., 2024). Synthetic polymers such as polyurethane, prized for its biocompatibility and malleability, are meticulously molded into intricate stent structures through advanced manufacturing techniques like injection molding or laser cutting, ensuring precise control over dimensions and mechanical properties critical for optimal performance within the dynamic cardiovascular system (Rapp et al., 2024). Similarly, biodegradable polymers like polylactic acid (PLA) undergo sophisticated processing methods to transform raw materials into stent scaffolds, leveraging techniques such as electrospinning or hot-melt extrusion to achieve the desired architecture and degradation kinetics. Polyethylene terephthalate (PET), renowned for its durability and stability, undergoes meticulous processing steps, including heat treatment and mechanical shaping, to yield stent frameworks capable of withstanding the mechanical stresses encountered during deployment within the arterial lumen. In contrast, natural polymers such as collagen, chitosan, and fibrin undergo specialized fabrication processes harnessing the inherent properties of biological materials, including techniques like solution casting, freeze-drying, or electrospinning, to create stent coatings or composite structures that seamlessly integrate with host tissues while promoting healing and regeneration (Lozano et al., 2022). As manufacturing technologies continue to evolve and converge, the fabrication of heart stents emerges as a harmonious symphony of material selection, design innovation, and precision engineering, driving forward the frontiers of cardiovascular medicine towards safer, more efficacious therapies for patients worldwide (Table 8).

**TABLE 8:** Aspects of fabrication and application of biomaterials for heart stents.

Properties	Synthetic polymers	Natural polymers
Biocompatibility	Generally high	Variable
Degradation Rate	Controllable, typically slow	Variable, can be fast or slow
Mechanical Strength	Tailorable, can be strong	Variable, may not be as strong as synthetics
Manufacturing Control	High, precise control over properties	Variable, may be influenced by source
Cost	Moderate to High, depending on material	Variable, depending on extraction/processing
Allergenic Potential	Low	Variable
Immunogenicity	Low	Variable
Thrombogenicity	Low	Variable
Antibacterial Properties	Can be engineered	Naturally present in some polymers
Surface Modification	Easily modified for specific functions	Limited modification potential
Radiopacity	Variable	Generally low
Processing Techniques	Melt Extrusion, Electrospinning, Injection Molding, 3D Printing	Solvent Casting, Freeze Drying, Crosslinking, Compression Molding, Sintering
Biodegradability	Typically, biodegradable	Naturally biodegradable
Materials	Polyurethane, Polycarbonate, Polyethylene, Polypropylene	Collagen, Chitosan, Fibrin, Silk Fibroin

## 7 Native extracellular matrix as cardiac scaffolding material

Proprietary extracellular matrix (ECM) can be synthesized using natural polymers. Cell cultures are established by using extracellular matrix (ECM) derived from animal sources and human donors. Stem cells (SCs) have the ability to undergo amplification, differentiation, survival, and phenotypic changes when exposed to natural extracellular matrix (ECM) ratios in a cultured environment (Liu et al., 2018). Previous investigations have demonstrated that contractile designed cardiac patches have the capability to undergo vascularization and intranasal administration, while also exhibiting *in vivo* survival for a duration of up to 8 weeks (Yao et al., 2019). Following their cultivation in extracellular matrix (ECM) mixes, the cells were then transplanted into Fischer 344 rats (Malandraki-Miller et al., 2018). Furthermore, the extracellular matrix (ECM) of isolated and modified hearts and other organs is utilized for the purpose of drug testing. In a study conducted by researchers, the impact of pharmacological treatments on myocardial, vascular-like structures, and intracellular Ca2+ transients were examined. This investigation involved the use of isolated rat hearts that were exposed to human cells in coronary arteries (Zarei et al., 2021). In summary, the extracellular matrix (ECM) within the cardiac tissue plays a crucial role in facilitating cellular proliferation, cell-specific differentiation, and the creation of myofilaments (Zhang et al., 2022). Tissue engineering (TE) might potentially get advantages from the utilization of the extracellular matrix (ECM) option, as it allows for the incorporation of synthetic biological materials and natural elements that can serve as improved substitutes for tissues, valves, or organs (Zhao et al., 2020) (Figure 3).

### 7.1 Investigating the application of native extracellular matrix in cardiac scaffold design

**FIGURE 5:**
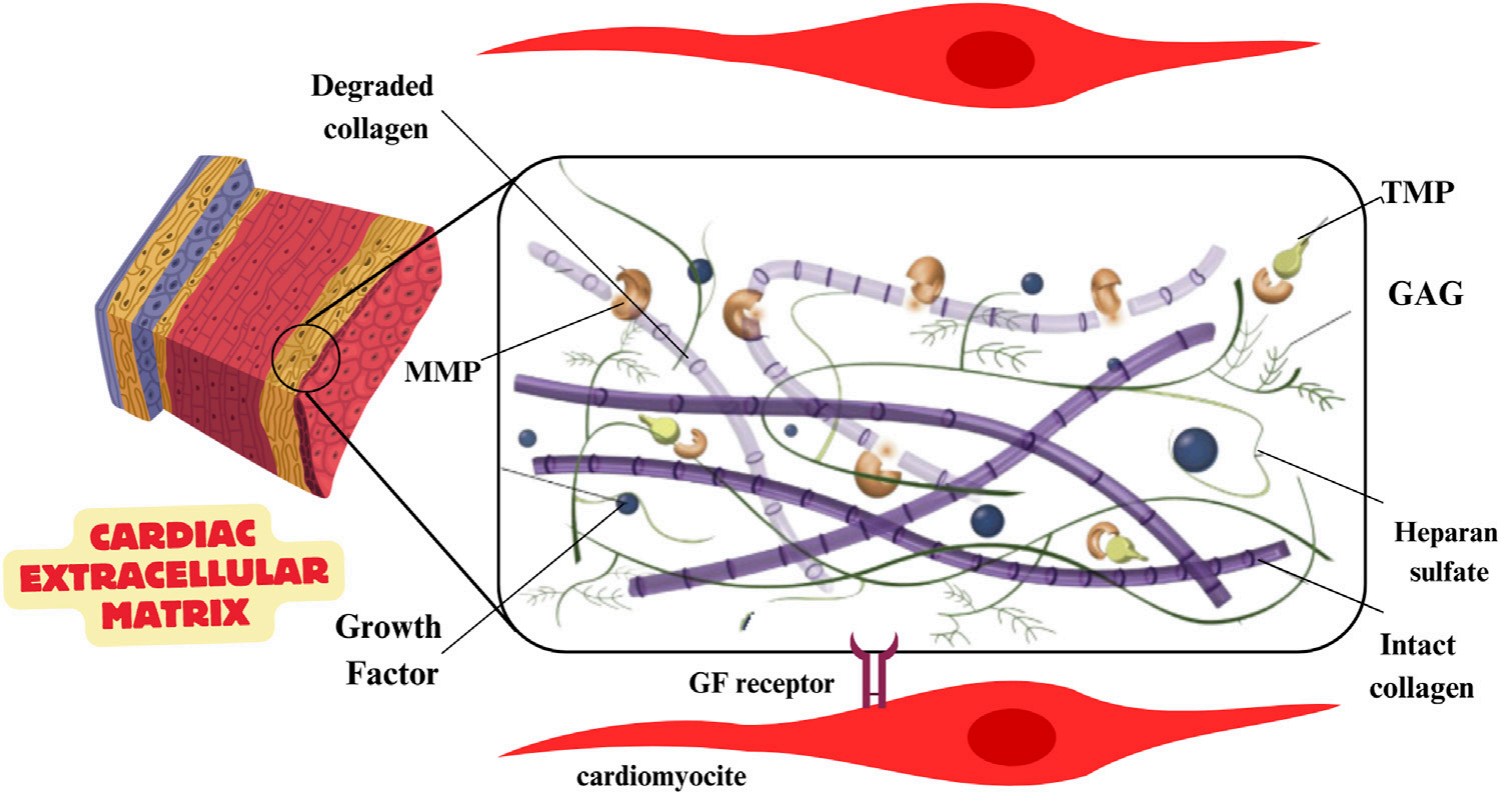
Cardiac extra cellular matrix and properties.

A potential strategy for tissue engineering involves designing cardiac scaffolds using native extracellular matrix (ECM) components. Biomaterials can be made more biocompatible by adding in components found in pericardial fluid, such as collagen types I, III, and IV, elastin, fibrin, and glycosaminoglycan (GAG) (Figure 5).

Regenerating functional organs is made possible with decellularized extracellular matrix (ECM) produced from heart tissue because it provides a non-immune environment with natural architecture and bioactive components. Possible cardiovascular uses for decellularized xenogeneic patches include pig small intestine submucosa (pSIS), which preserves the native ECM architecture, induces tissue regeneration, and reduces host immunological reactions (Sönmezer et al., 2023). Decellularized cardiac matrix, created by combining sodium dodecyl sulphate (SDS) with Triton X-100, is an excellent scaffold for tissue engineering since it maintains the structure and protein content of the extracellular matrix (ECM). By creating an ideal setting for cell attachment, proliferation, and differentiation, pericardial ECM can help regenerate myocardium after myocardial infarction. Enhancing cellular responsiveness and tissue regeneration, native extracellular matrix (ECM) inclusion into cardiac scaffolds has a substantial impact (Liu et al., 2024). It is essential for the tissue’s macro- and microstructure to be preserved during regeneration, and native ECM offers this non-immune milieu with bioactive components. In particular, extracellular matrix (ECM) sourced from the human pericardium facilitates cell adhesion, proliferation, and differentiation, all of which aid in the recovery of damaged heart tissue following a myocardial infarction. Incorporating polyaniline and other materials into scaffolds improves electrical conductivity, which in turn promotes cardiomyocyte regeneration and may help heal heart damage (Wang et al., 2017). Roshanbinfar K. et al. (Roshanbinfar et al., 2018) have developed a biohybrid hydrogel using collagen, alginate, and electroconductive poly (3,4-ethylenedioxythiophene):polystyrene sulfonate (PEDOT:PSS). This innovative hydrogel showcases fibrous structures mimicking the extracellular matrix, along with enhanced electrical coupling and improved cardiomyocyte maturation. The inclusion of PEDOT:PSS in the hydrogel not only enhances electrical conductivity but also acts as a preventive measure against arrhythmia in tissue constructs containing neonatal rat cardiomyocytes. Improvements in tissue-biomaterial interactions and therapeutic results are made possible by these advances in biomaterial engineering, which permit the tailoring of mechanical strength, bioactivity, and electroconductivity as features of scaffolds (Zimmermann et al., 2010; Ye et al., 2013; Esser and Engel, 2021; Barbulescu et al., 2022; Zhang et al., 2022). A number of investigations have investigated cardiac tissue engineering using decellularized extracellular matrix (dECM) harvested from myocardial tissue. Cardiac dECM has distinct benefits over synthetic biomaterials, as shown by Crapo et al., including the ability to retain biochemical signals that promote cell adhesion, migration, differentiation, and proliferation; mechanical properties that mimic those of the surrounding tissue; and preservation of the microstructure and composition of the ECM specific to the organ (Golebiowska et al., 2024).

Using cardiac-specific markers as evidence, Rajabi and colleagues developed “rat hearts with human characteristics” by introducing cardiac progenitor cells derived from human embryonic stem cells (ESC) into scaffolds stripped of cellular material. The addition of bFGF during perfusion enhanced the CPCs’ retention and their transformation into cardiomyocytes, smooth muscle cells, and endothelial cells (Rajabi et al., 2018). Similarly, Guyette et al. (2016). demonstrated that cardiac cells produced from human induced pluripotent stem cells could be seeded onto decellularized myocardium tissue from rats. These cells then formed functional patches that, when implanted into a rat model of myocardial infarction, improved heart function. Badylak (2002). pointed out that there are still several obstacles to overcome in the current methods of decellularization and recellularization of dECM. These include achieving prevascularization of thick dECM constructs, optimizing the material properties of dECM to increase bioactivity, and achieving efficient recellularization of dECM to achieve a homogenous cell distribution. Ott et al. (2008). are currently working to overcome these obstacles and enhance the effectiveness of cardiac dECM scaffolds in regenerating and repairing myocardial tissue.

## 8 Conclusion

Despite significant advancements in restoring heart function, cardiovascular disease remains the leading cause of mortality globally. One of the primary challenges is the inability of injured cardiomyocytes to self-renew, leading to terminal differentiation in dysfunctional hearts or replacement with fibrotic scar tissue. Moreover, therapies such as heart transplants and ventricular support devices face limitations due to side effects of immunosuppressive drugs and a shortage of donors. Moving forward, the field of cardiac tissue engineering holds immense promise. However, several challenges need to be addressed. While synthetic biomaterials have been utilized for cardiac tissue repair, none have accurately replicated the native tissue architecture. Issues such as inadequate cell migration, limited clinical applicability, and immune-related complications hinder their effectiveness. An emerging approach involves leveraging natural biomaterials derived from extracellular matrix (ECM). ECM-based biologic scaffolds, stripped of cellular contents and antigenic epitopes, retain the mechanical stability and properties of native tissue, offering a promising avenue for cardiac tissue regeneration. Future directions in the field should focus on refining biomaterial design to mimic the intricate structure of native cardiac tissue, promoting cell migration and integration, and enhancing immunomodulatory properties to mitigate immune-related challenges. Additionally, advancements in tissue engineering techniques, such as 3D printing, can facilitate the fabrication of complex cardiac constructs with precise control over structure and composition. Overall, by overcoming these challenges and exploring innovative approaches, cardiac tissue engineering holds the potential to revolutionize the treatment of cardiovascular disease, offering hope for improved patient outcomes and quality of life.

## Abbreviations

MI, Myocardial Infarction; CVD, Cardiovascular Disease; AMI, Acute Myocardial Infarction; CMs, Cardiomyocytes; HF, Heart Failure; BVS, Bioresorbable Vascular Scaffolds; ECM, Extracellular Matrix; SCs, Stem Cells; BMMNCs, Bone Marrow Mononuclear Cells; CMs, complementary metal-oxide semiconductors; PFC, perfluorocarbon oxygen compound; bFGF, basic fibroblast growth factor; dECM, decellularized extracellular matrix; ECM, extracellular matrix.
