# Supplementary figures and images for: Advancements in tissue engineering for cardiovascular health: a biomedical engineering perspective
Source: Front Bioeng Biotechnol. 2024 May 31;12:1385124. doi: 10.3389/fbioe.2024.1385124 (PMC11176440; doi:10.3389/fbioe.2024.1385124)

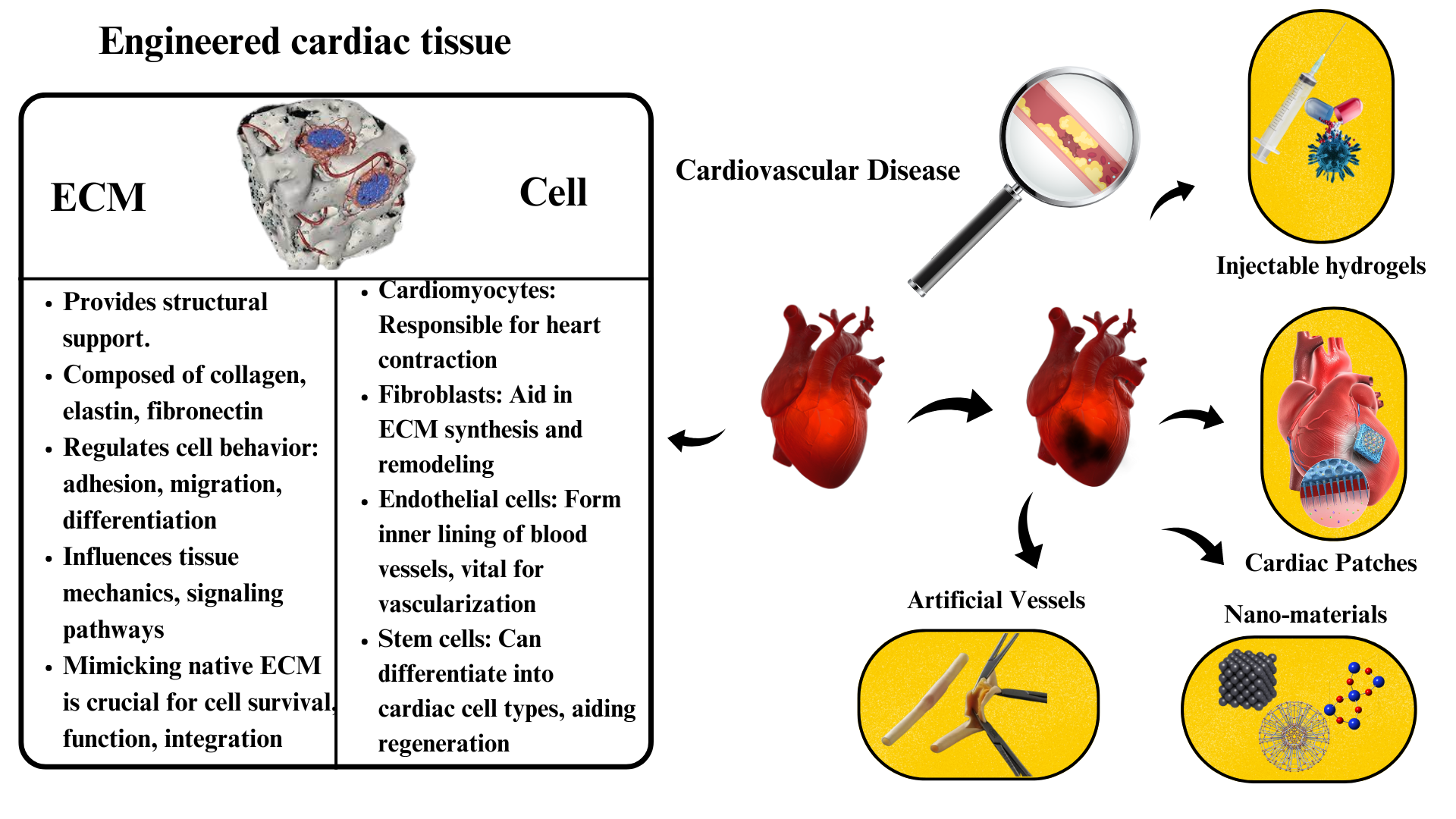

Supplement: Supplementary file 1 [file Image5.PNG]

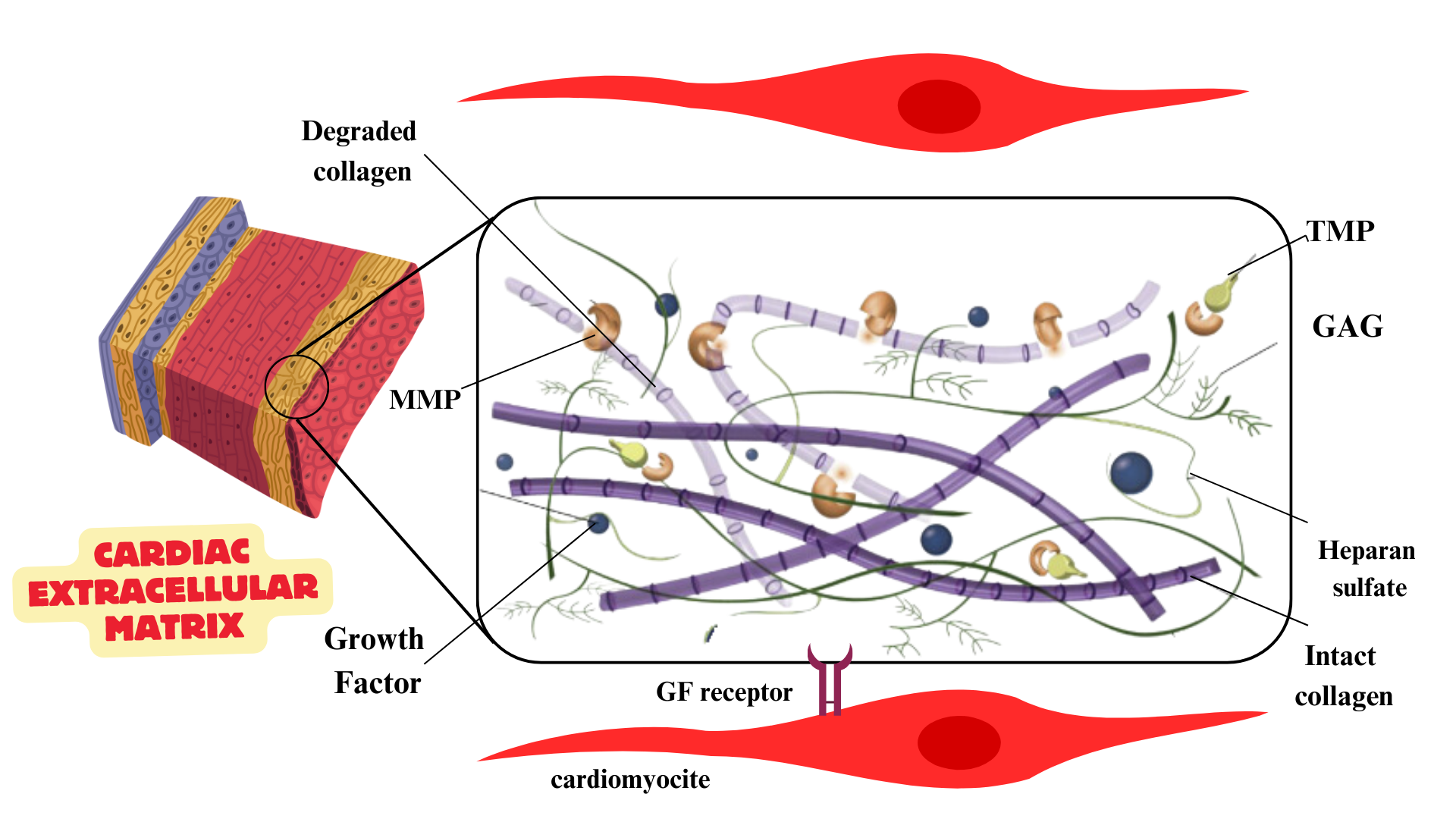

Supplement: Supplementary file 2 [file Image4.PNG]

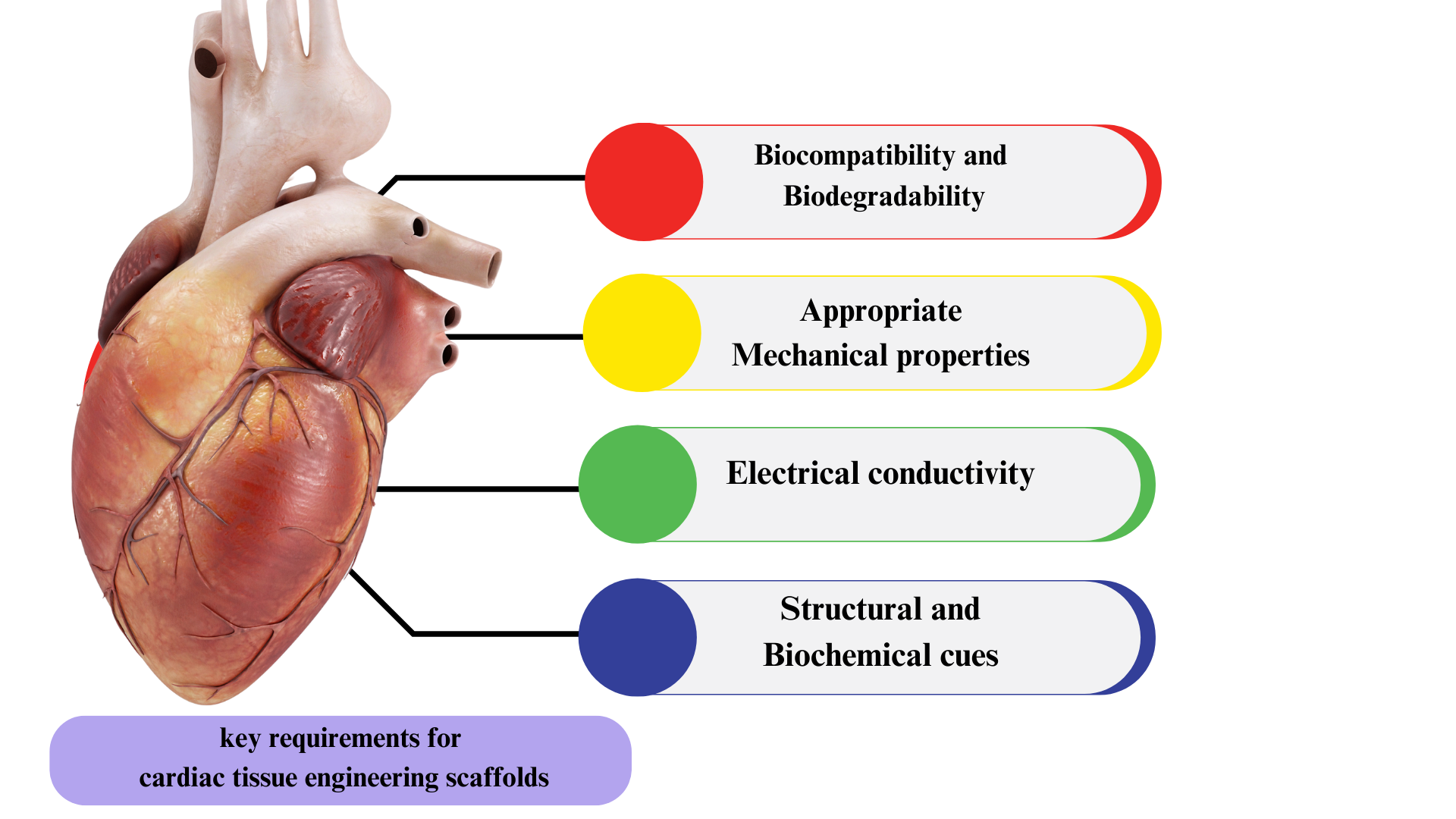

Supplement: Supplementary file 3 [file Image2.PNG]

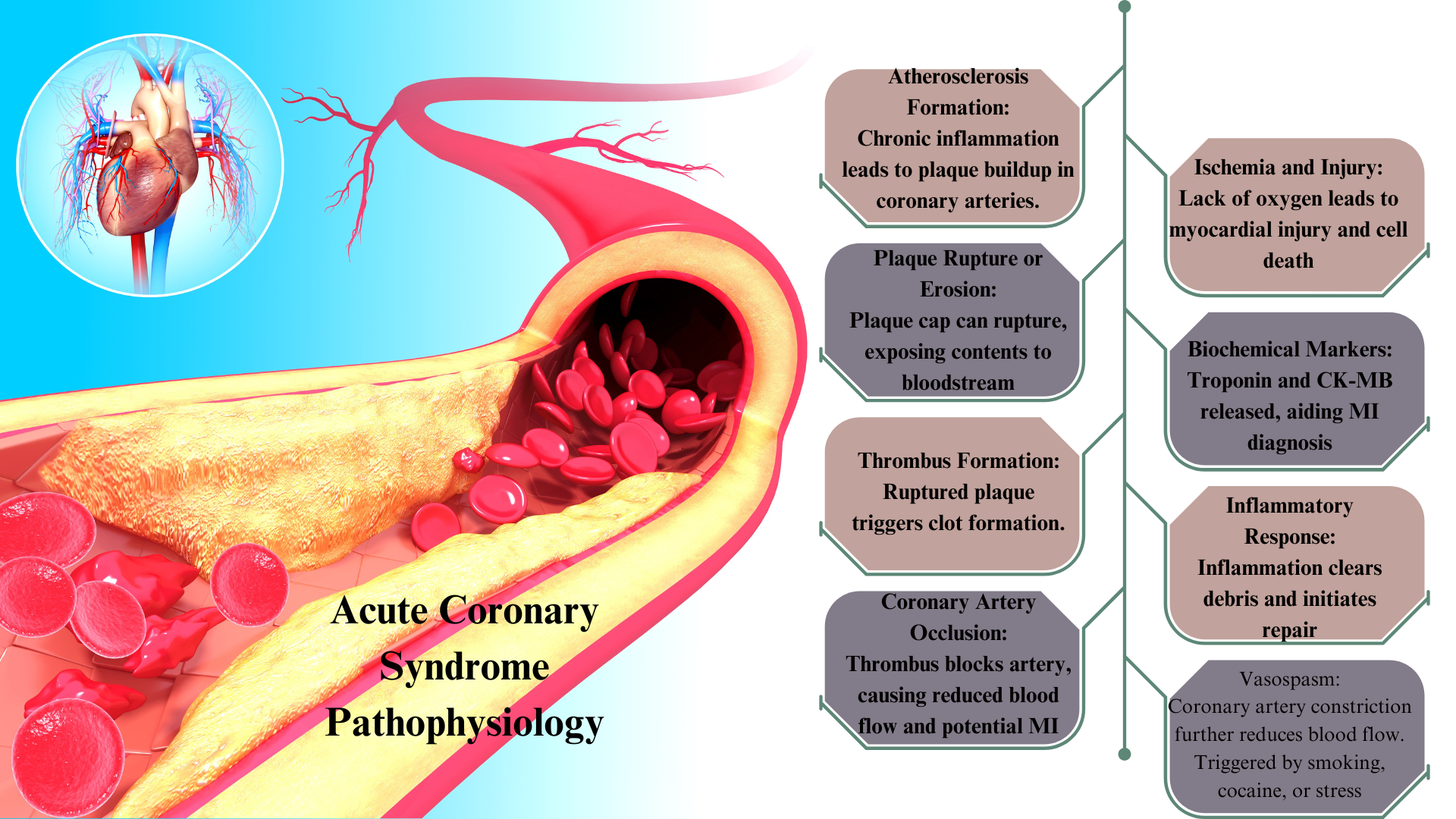

Supplement: Supplementary file 4 [file Image1.PNG]

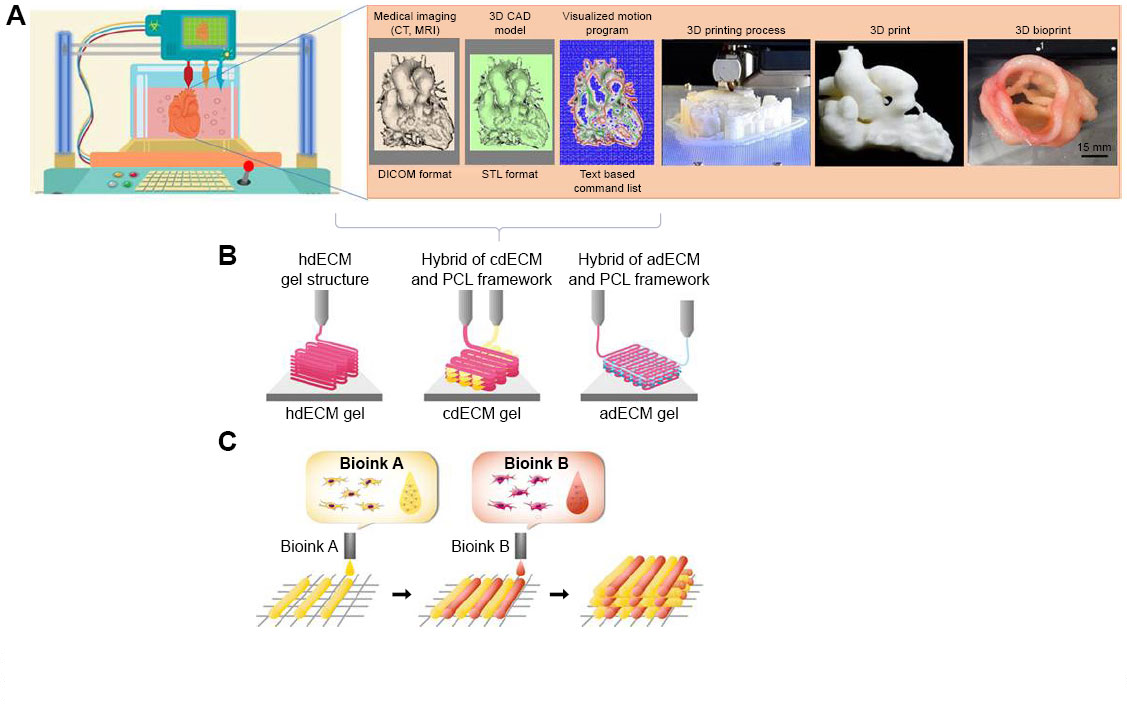

Supplement: Supplementary file 5 [file Image3.PNG]
